# Mitochondrial dysfunction is associated with lipid metabolism disorder and upregulation of angiotensin-converting enzyme 2

**DOI:** 10.1371/journal.pone.0270418

**Published:** 2022-06-29

**Authors:** Qian Zhao, Xiaoshan Zhou, Raoul Kuiper, Sophie Curbo, Anna Karlsson

**Affiliations:** 1 Division of Clinical Microbiology, Department of Laboratory Medicine, Karolinska Institute, Karolinska University Hospital, Stockholm, Sweden; 2 Division of Pathology, Department of Laboratory Medicine, Karolinska Institute, Karolinska University Hospital, Stockholm, Sweden; 3 Norwegian Veterinary Institute (Elizabeth Stephansens vei 1, 1433 Ås, Norway Section for Aquatic Biosafety), Oslo, Norway; University of Houston, UNITED STATES

## Abstract

Thymidine kinase 2 (TK2) deficiency in humans leads to a myopathic form of mitochondrial DNA (mtDNA) deficiency. Here we present a skeletal and cardiac muscle specific TK2 knockout mouse (mTk2 KO). The mice showed dilated hearts and markedly reduced adipose tissue during week 12 to 16. A severe decrease of mtDNA was found only in skeletal muscle and heart tissue in mTk2 KO mice. Expression analysis of key metabolic genes of 16 weeks knockout mice showed significant changes of genes involved in lipid metabolism, with different patterns in heart and skeletal muscle. Our study further suggests that lipoprotein lipase (LPL) from liver supports the metabolism when heart and skeletal muscle were impaired due to mitochondrial dysfunction. The angiotensin-converting enzyme 2 (ACE2), which is involved in glucose homeostasis, was also affected by mtDNA deficiency in our study. Interestingly, both the gene and protein expression of ACE2 were increased in cardiac tissue of mTk2 KO mice. Since ACE2 is a receptor for the SARS-CoV-2 virus, its regulation in relation to mitochondrial function may have important clinical implications.

## Introduction

Thymidine kinase 2 (TK2) is a pyrimidine deoxyribonucleoside kinase that phosphorylates dThd, dUrd and dCyd to their respective monophosphates [1]. TK2 is a nuclear encoded enzyme with an N-terminal mitochondrial targeting signal, and it is constitutively expressed throughout the cell cycle. Patients with TK2 mutations have a myopathic form of mitochondrial DNA (mtDNA) depletion syndrome (MDS), with short life expectancy and with predominant symptoms of hypotonia and muscle weakness [2]. More specifically, TK2 deficiency results in low levels of mtDNA, that encodes for proteins in the electron transport chain and subsequently exerts effects on ATP production.

Genetic defects in any of the genes involved in mitochondrial nucleotide synthesis, mtDNA replication or mitochondrial function may result in pathology caused by mitochondrial deficiency [3]. Moreover, mitochondria are involved in diverse conditions that are not directly related to deficiency of mitochondrial function, such as the metabolic reprogramming observed in cancer [4] and mice with mtDNA deficiency have been demonstrated with a phenotype of premature aging affecting the entire animal [5]. Muscular dysfunction is commonly associated with mitochondrial disorders. In addition to the function of muscle to make movement possible, muscle tissue is also metabolically active as a primary site for uptake and storage of glucose and a reservoir of amino acids [6]. Both skeletal and cardiac muscle adapt in response to conditions such as illness and aging, and to counteract a muscular decline is regarded as beneficial to health [7].

We have previously studied a complete TK2 knockout mouse model [8]. This model showed a severe phenotype with multi organ abnormalities and a short survival of 2–3 weeks. To further study mechanisms and possible treatment strategies of the myopathic form of MDS, we now have generated a heart and skeletal muscle specific TK2 knockout mouse model. Our aim was to develop a TK2 mouse model with a prolonged life span to enable both mechanistic and intervention studies. In addition to mtDNA deficiency in the targeted tissues, skeletal muscle and heart, the most obvious phenotype of the mTk2 KO mice was an almost complete loss of adipose tissue. With the observed phenotype we investigated alterations in fat and energy metabolism with focus on gene expression in heart and skeletal muscle. Our results show alterations of genes encoding key enzymes related to mitochondria and lipid metabolism. Since depletion of the angiotensin- converting enzyme 2 (ACE2) in mice has suggested that ACE2 is involved in regulation of the blood glucose level [9], we investigated the effect of mitochondrial dysfunction on the ACE2 gene and protein expression in heart and skeletal muscle. Interestingly, the expression of ACE2, the main receptor for SARS-CoV-2, was highly augmented in cardiomyocytes, but not in skeletal muscle, in our mTk2 KO mice model. We also detected an increased level of the lipoprotein lipase (Lpl) protein in heart, skeletal muscle, and liver, possibly related to the consumption of overall body fat. The mitochondrial dysfunction in heart tissue caused damaged heart muscle cells and dilated hearts that probably contributed to the observed sudden death of the mice at around 16 weeks of age.

We here present a model of muscle specific depletion of mtDNA that in addition to muscular weakness also shows severe metabolic alterations. The phenotype of the mTk2 KO mice demonstrate the importance of both skeletal and heart muscle for the metabolic homeostasis of the mice and the specific involvement of mitochondria in these tissues to efficiently balance and store energy in adipose tissue.

## Results

### Characterization of the mTK2 KO mice

**Fig 1:**
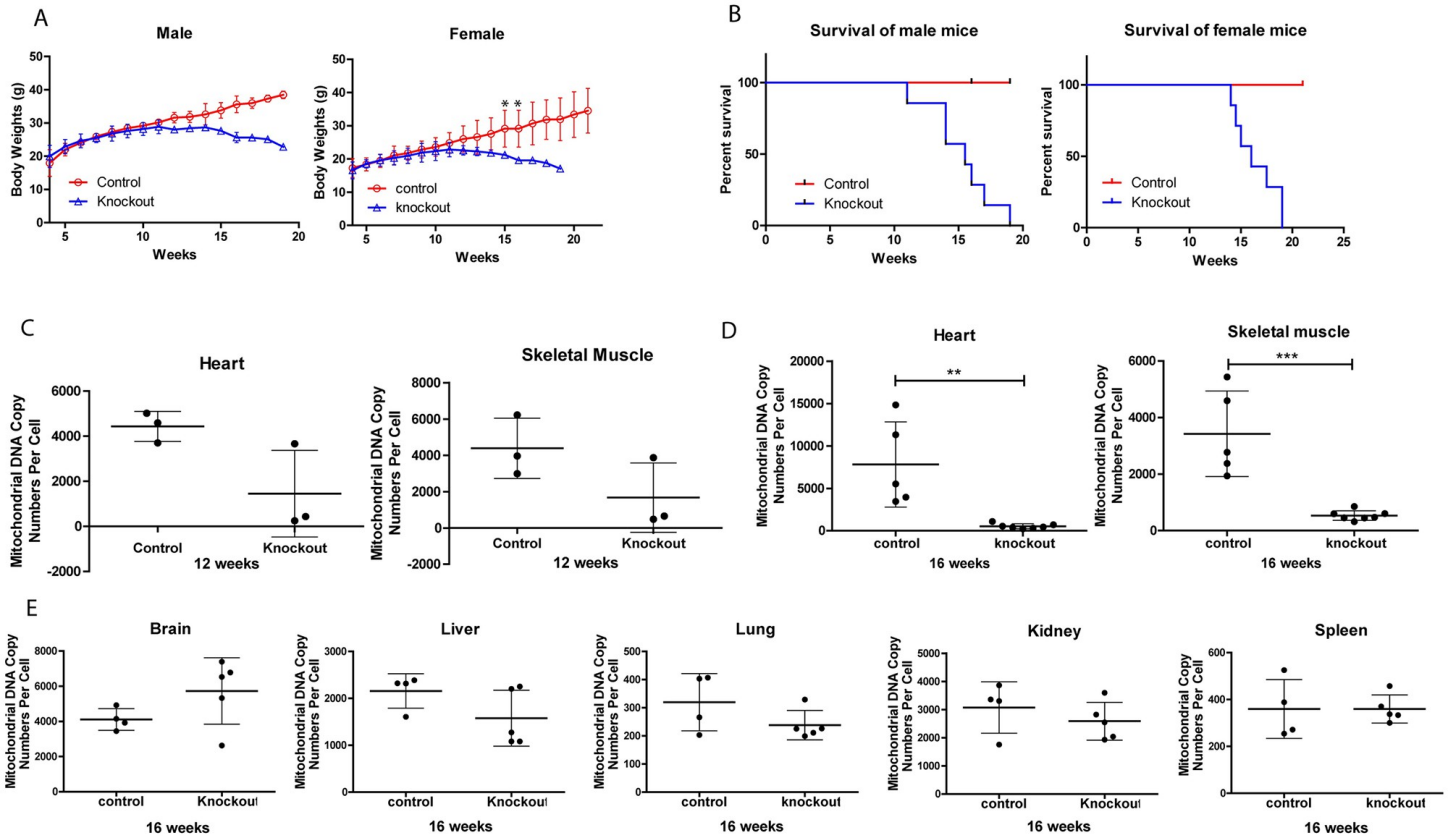
Life expectancy, body weight and mtDNA copy number. (A) Weight of male and female mice between 4 and 20 weeks of age. n = 3 mice per group of male, and n = 5 mice per group of female. (B) Life span of male and female mice between 4 and 20 weeks of age. n = 7 mice per group. (C) mtDNA copy number of 12 weeks mice heart and skeletal muscle. (D) 16ax weeks heart and skeletal muscle. (E) Other important organs in 16 weeks mice did not show mtDNA deficiency symptom. Data are shown as means ± S.D. For (C-E), n = 3–8 mice per group. For (A,C-E), groups were compared using the Mann-Whitney *U*-test. Significant levels were set to p < 0.05(*), p < 0.01(**), p < 0.001(***).

We first generated the skeletal and cardiac muscle specific TK2 knockout mice (S1 A, B), and tested the tissue specificity (S1 E). The most obvious symptom of the mTk2 KO mice, as compared to control mice, was a body weight decrease starting at 12 weeks of age. Both male and female mice became very slim within the following 4–6 weeks (Fig 1A). The male mice behaved normally although 6 out of 7 mice died suddenly and unexpectedly at week 12–16, probably due to acute heart failure. One male mouse was sacrificed at week 19 due to loss of weight. Of the female mice, 4 out of 7 died suddenly at 13–19 weeks of age while 3 were sacrificed at 15–19 weeks of age due to weight loss. The average lifespan of the male knockout mice was slightly shorter (15.2 weeks) as compared to the female knockout mice (16.4 weeks) (Fig 1B). Based on these observations, samples of 12- and 16-weeks old mice were analyzed for mtDNA levels, histopathology, and gene expression. At 12 weeks there was no statistical difference in mtDNA levels in heart and skeletal muscle of the control and knockout mice, although the averages of the mtDNA levels were lower in the mTk2 KO mice (Fig 1C). At 16 weeks, the mtDNA levels of heart and skeletal muscle tissue were significantly lower in the mTk2 KO mice compared to the control group (Fig 1D). In brain, liver, lung, kidney, and spleen there were no differences in the mtDNA copy numbers between the two groups of 16 weeks old mice (Fig 1E).

### Muscle specific knockout of TK2 leads to low fat deposits

**Fig 2:**
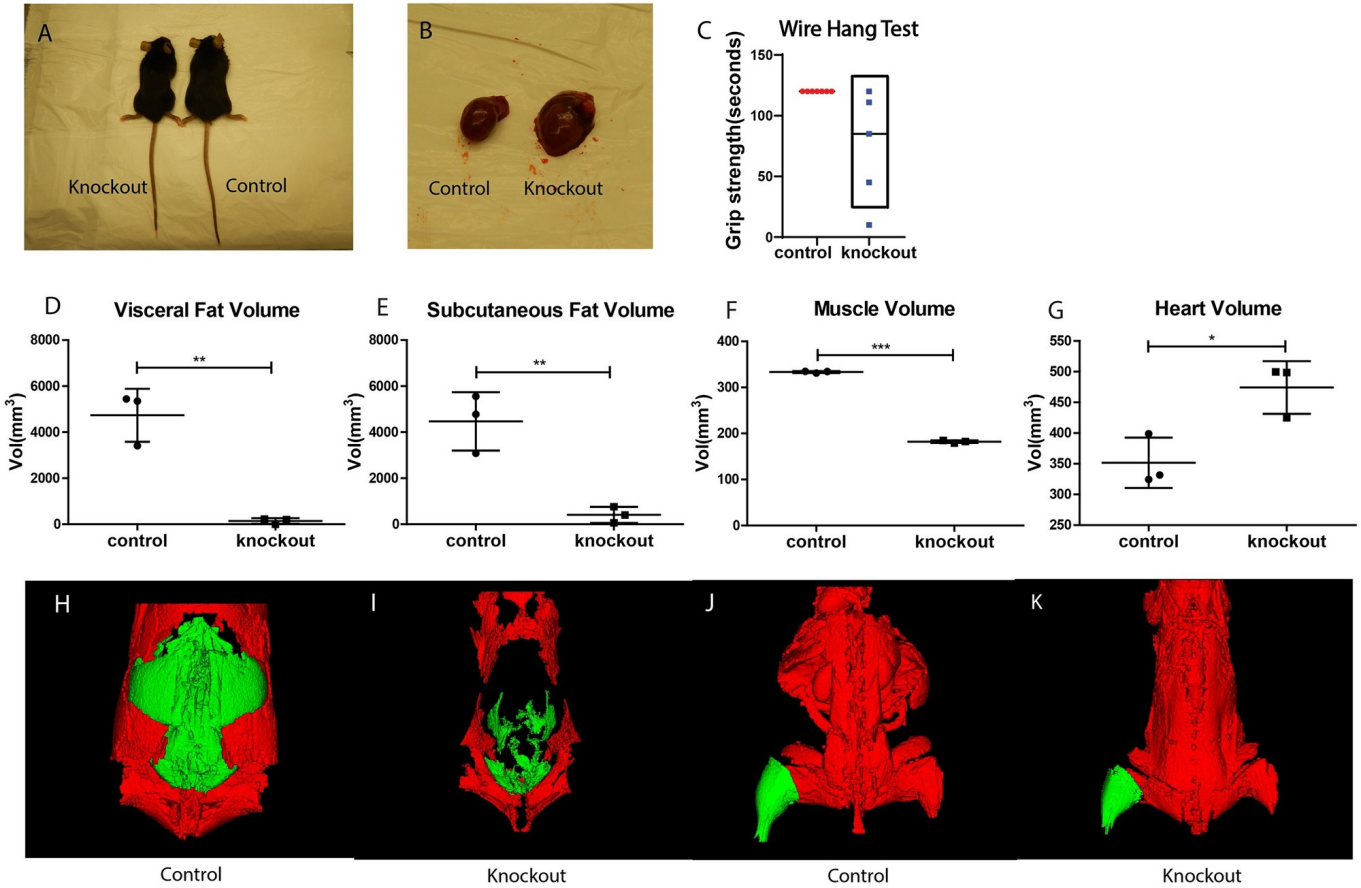
Fat and muscle volume. (A-B) Picture of control and knockout mice (A), and heart (B). (C) Wire hang test results differs around 16 weeks. n = 5 mice per group. Visceral fat (D), subcutaneous fat (E), leg muscle volume (F) and heart volume (G) of control and knockout mice. n = 3 mice per group. Data are shown as means ± S.D. Groups were compared using the Mann-Whitney *U*-test. (H-K) CT scan of fat and muscle volume, visceral fat showed in green and subcutaneous fat in red (H-I), and leg muscle in green (J-K). All the data were taken from 16 weeks mice. Significant levels were set to p < 0.05(*), p < 0.01(**), p < 0.001(***).

The mTk2 KO mice were relatively leaner compared to the control mice as observed by eye (Fig 2A). The distribution of adipose tissue was investigated by CT scan, where the visceral and subcutaneous fat volumes were calculated. The results showed that the mTk2 KO mice had extremely little of both visceral (Fig 2D and 2I) and subcutaneous fat (Fig 2E and 2I) compared to control mice (Fig 2H). The body weight curves of mTk2 KO showed significant deviation from the controls between age week 12 and 18. During this time the control mice gained weight every day whereas the knockout mice continuously lost body weight (Fig 1A). The muscle volume was also investigated by measurement of the leg as a representative part. The average leg of the mTk2 KO mice contained less muscle tissue and was only about two thirds the size of the average hind leg muscle of the control mice (Fig 2F, 2J and 2K). The mTk2 KO group at around 16 weeks had lower muscle strength than the control group according to a wire hang test, with an average time of 74.5 s in the knockout group as compared to the max length of 120 s for the control group (Fig 2C). The heart volume of the mTk2 KO mice was increased in the 16 weeks old mice (Fig 2B and 2G) and showed thinner heart walls when examined, suggesting that the heart was dilated in knockout mice. Inspection of the mTk2 KO mice confirmed that they had healthy teeth but there is no food intake data available.

### Histopathology of muscle specific TK2 knockout mouse tissues

**Fig 3:**
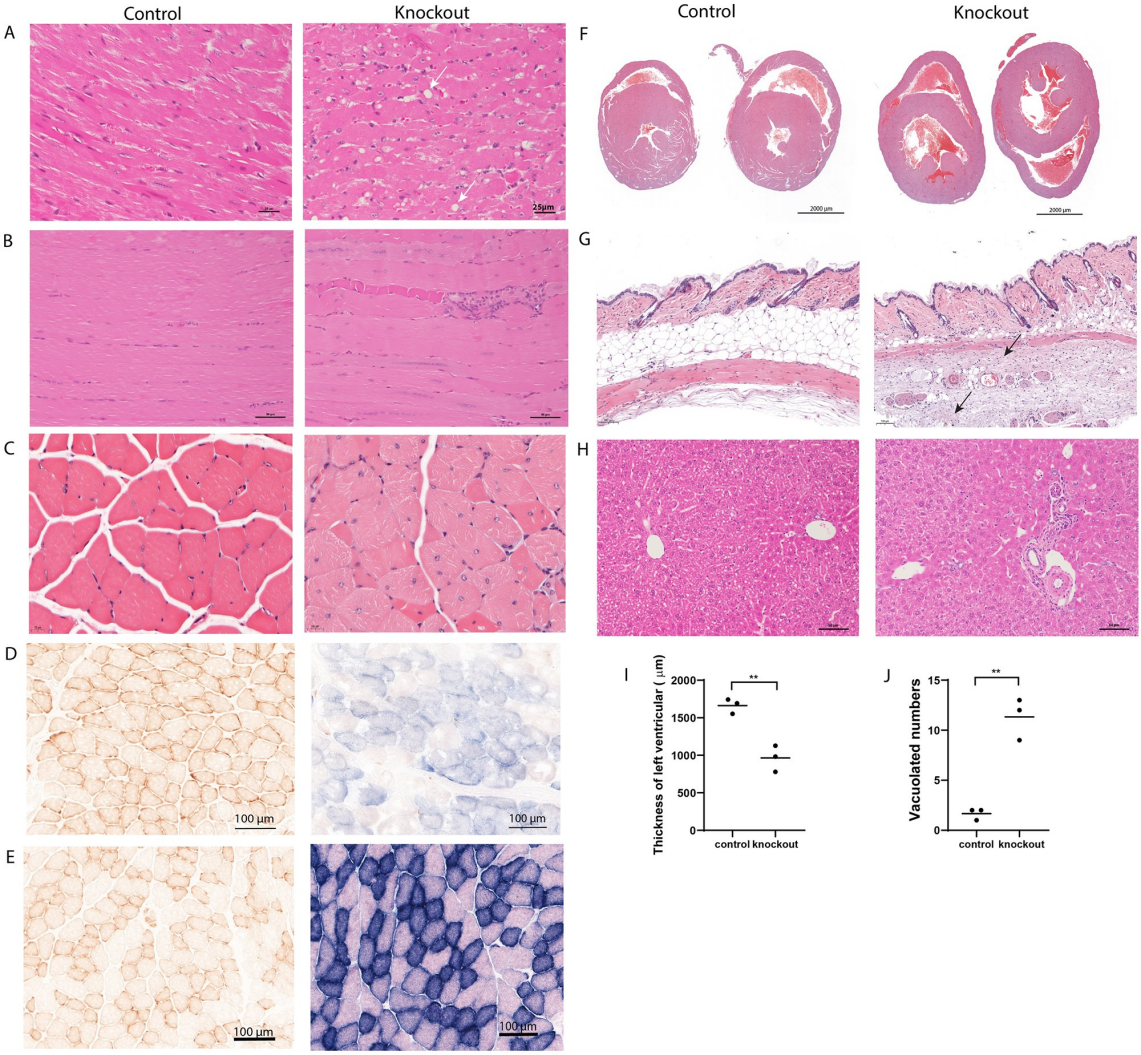
Histology and enzyme activity. (A-C, F-H) Histology results from 16 weeks mice, cardiac muscle (A), skeletal muscle (B), transverse sections of skeletal muscle (C), horizontal view of heart (F), skin (G), and liver (H). COX/SDH staining of control and knockout mice skeletal muscle (D), COX staining control and SDH staining control (E), thickness of left ventricular of control and knockout mice (I), vacuolated numbers in view of 40X of control and knockout mice (J). For (A-J), n = 3 mice per group.

Both skeletal muscle and heart muscle showed histopathological changes in mTk2 KO mice. The major alteration of the heart muscle was the thinner cardiac wall (Fig 3F and 3I), but heart muscle of mTk2 KO mice also showed vacuolated cardiomyocytes (Fig 3A and 3J). The skeletal muscle of mTk2 KO mice showed thinner muscle fibers, centralization of nuclei, as compared to the control mice (Fig 3B and 3C). Cryosections of the skeletal muscle of control mice were all saturated by DAB brown color staining illustrating a strong activity of mitochondrial complex IV, and the blue color representing complex II was overlapped by the brown color of control mice (Fig 3D). However, in 16 weeks old mTk2 KO mice muscle fibers, the activity of the mitochondrial complex IV was decreased, as shown by the mixed pattern (brown and blue) in the image of knockout mice (Fig 3D). COX and SDH staining control are shown in Fig 3E. The results in mTk2 KO mice demonstrated a good functional activity of mitochondrial complex II but impaired enzyme activity of complex IV (Fig 3D).

The panniculus carnosus muscle of the skin of the mTk2 KO mice was damaged and showed slight fibrosis, as compared to the control mice (Fig 3G). The subcutaneous fat layer almost disappeared completely in the mTk2 KO mice (Fig 3G), consistent with the weight curve and the CT scan results. The liver tissue showed minor changes with signs of moderate portal fibrosis in the mTk2 KO mice (Fig 3H).

### Altered expression of genes in lipid metabolism in heart, skeletal muscle, and liver tissue

**Fig 4:**
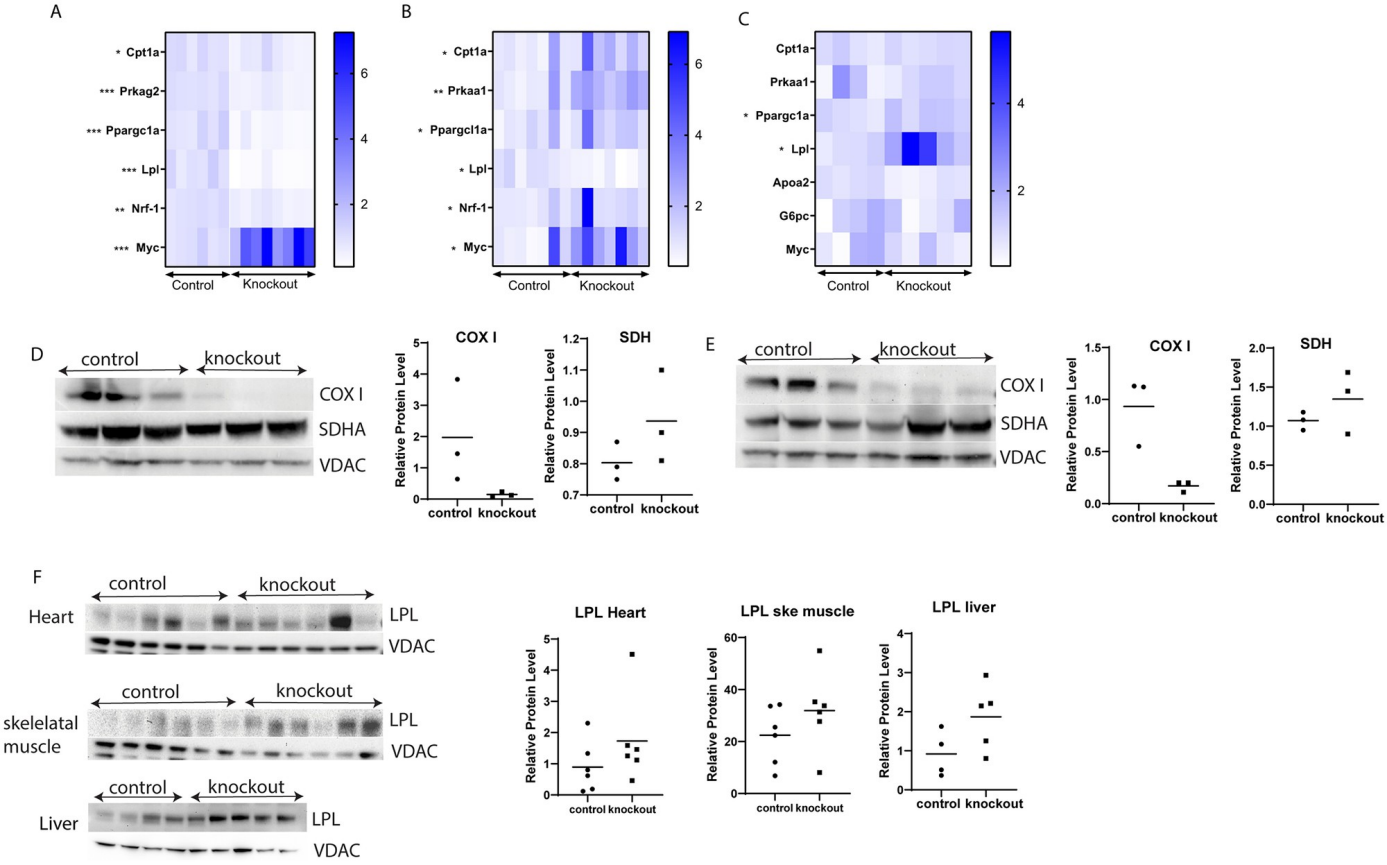
Gene expression in 16 weeks old Tk2 KO mice. (A) 16 weeks mice heart gene expression. n = 6 mice for control group, and n = 8 mice for knockout group. (B) 16 weeks mice skeletal muscle gene expression. n = 7 mice per group. (C) 16 weeks mice liver gene expression. n = 4 mice for control group, and n = 5 mice for knockout group. For (A-C), groups were compared using the Mann-Whitney *U*-test. (D) 16 weeks mice heart COX I and SDH protein level. (E) 16 weeks mice skeletal muscle COX I and SDH protein level. For (D-E), n = 3 mice per group. (F) 16 weeks mice heart and skeletal muscle LPL protein level. n = 4 mice for control group, and n = 5 mice for knockout group. For (A-F), groups were compared using Mann-Whitney *U*-test. Significant levels were set to p < 0.05(*), p < 0.01(**), p < 0.001(***).

Based on the observed phenotype and online database analysis results (S 2), expression of 17 key enzymes for fatty acid oxidation and genes targeting energy production and catabolism were analyzed. There were no changes when testing 12 weeks heart tissue and skeletal muscle (S3A and S3C Fig). Both similarities and differences between skeletal muscle and heart muscle were found when tissues were analyzed at 16 weeks of age, although most of the genes analysed were altered in heart tissue (S3B Fig). One major alteration was observed for the *c-myc* oncogene, a transcription factor that activates genes involved in mitochondrial function and metabolism, that showed 4.6 times higher expression in mTk2 KO mice in heart tissue (Fig 4A). A similar picture with increased *c-myc* expression was found also in skeletal muscle of 16 weeks old mTk2 KO mice but not in liver tissue (Fig 4B and 4C).

Another gene showing altered expression in heart and skeletal muscle was the gene encoding lipoprotein lipase, *Lpl*. The expression of the *Lpl* gene was significantly decreased in both skeletal muscle and heart muscle in 16 weeks old mTk2 KO mice. In contrast, the expression of the *Lpl* gene was increased in liver tissue (Fig 4C). LPL is known to differ in expression related to the status of fatty acid metabolism and to provide fatty acids as an energy source. This interesting observation prompted us to analyze LPL also at the protein level (Fig 4F). Although differences were not statistically significant, the average levels were increased in all tissues investigated; heart, skeletal muscle and liver.

AMP-activated protein kinase (AMPK) is an important energy sensing enzyme with different catalytic and regulatory subunits encoded by different genes. The regulatory AMPK subunit gamma-2 is an enzyme encoded by the *Prkag2* gene and was decreased in heart muscle (Fig 4A), while this gene is not expressed in skeletal muscle. Another subunit of AMPK, the catalytic subunit alpha-1, encoded by the *Prkaa1* gene, was upregulated in skeletal muscle (Fig 4B) while not expressed in heart tissue.

Another master energy regulator is peroxisome proliferator-activated receptor gamma coactivator 1-alpha (PGC-1α), which is a protein that is encoded by the *Ppargc1a* gene. PGC-1α is a regulator of mitochondrial biogenesis and PGC-1α is also regulating liver gluconeogenesis by inducing increased gene expression for gluconeogenesis [10]. The gene encoding PGC-1α showed significantly lower expression in heart tissue (Fig 4A) but was upregulated in skeletal muscle (Fig 4B) of mTk2 KO mice compared to control mice at 16 weeks of age. The *Ppargc1a* gene was upregulated also in liver and showed 1.4 times higher expression, as compared to the control group (Fig 4C). The nuclear respiratory factor 1 (*Nrf1*) and carnitine palmitoyltransferase (*Cpt1a*) were regulated by PGC-1α [11]. Nuclear respiratory factor 1 is a transcription factor that activates the expression of key metabolic genes regulating cellular growth and mitochondrial DNA transcription and replication. Also, *Nrf1* was upregulated in skeletal muscle (Fig 4B) while downregulated in heart muscle (Fig 4A) of the analyzed mTk2 KO mice. A similar situation was found for *Cpt1a*, a mitochondrial enzyme associated with the outer mitochondrial membrane. *Cpt1a* has a role in fatty acid metabolism and beta-oxidation of log chain fatty acids. Expression of this gene was increased in skeletal muscle (Fig 4B), decreased in heart muscle (Fig 4A) and with no change in liver tissue (Fig 4C). The expression of other investigated genes in 16 weeks heart tissue is shown in the supplementary data (S.3B).

We also investigated the COX I and SDH protein level in heart and skeletal muscle of 16 weeks old mice, and the results were consistent with the in situ activities of COX I and SDH. Both heart and skeletal muscle showed high COX I protein level in the control group and very low COX I protein level in mTk2 KO mice (Fig 4D and 4E). Heart and skeletal muscle had similar SDH protein levels when control and knockout groups were compared (Fig 4D and 4E).

Liver tissue of mTk2 KO mice retained normal mtDNA levels and the histopathology results showed only minor differences compared to the control group. Since we were interested in gene alterations affecting cell metabolism and energy production, we investigated liver tissue despite the normal mtDNA levels. We analyzed the glucose-6-phosphatase catalytic subunit (*G6pc*) gene, which is a key gene related to gluconeogenesis, but the *G6pc* gene did not change its expression in mTk2 KO mice compared to the control group (Fig 4C). The expression of *c-myc*, *Cpt1a*, and *Prkaa1* were unaltered as well (Fig 4C). The largest upregulated gene was *Lpl*, where the mTk2 KO mice showed 3 times higher expression level in liver as compared to the control group, which indicated liver involvement in the altered metabolism with the complete loss of adipose tissue (Fig 4C).

### The expression of ACE2 in skeletal muscle and heart tissue

**Fig 5:**
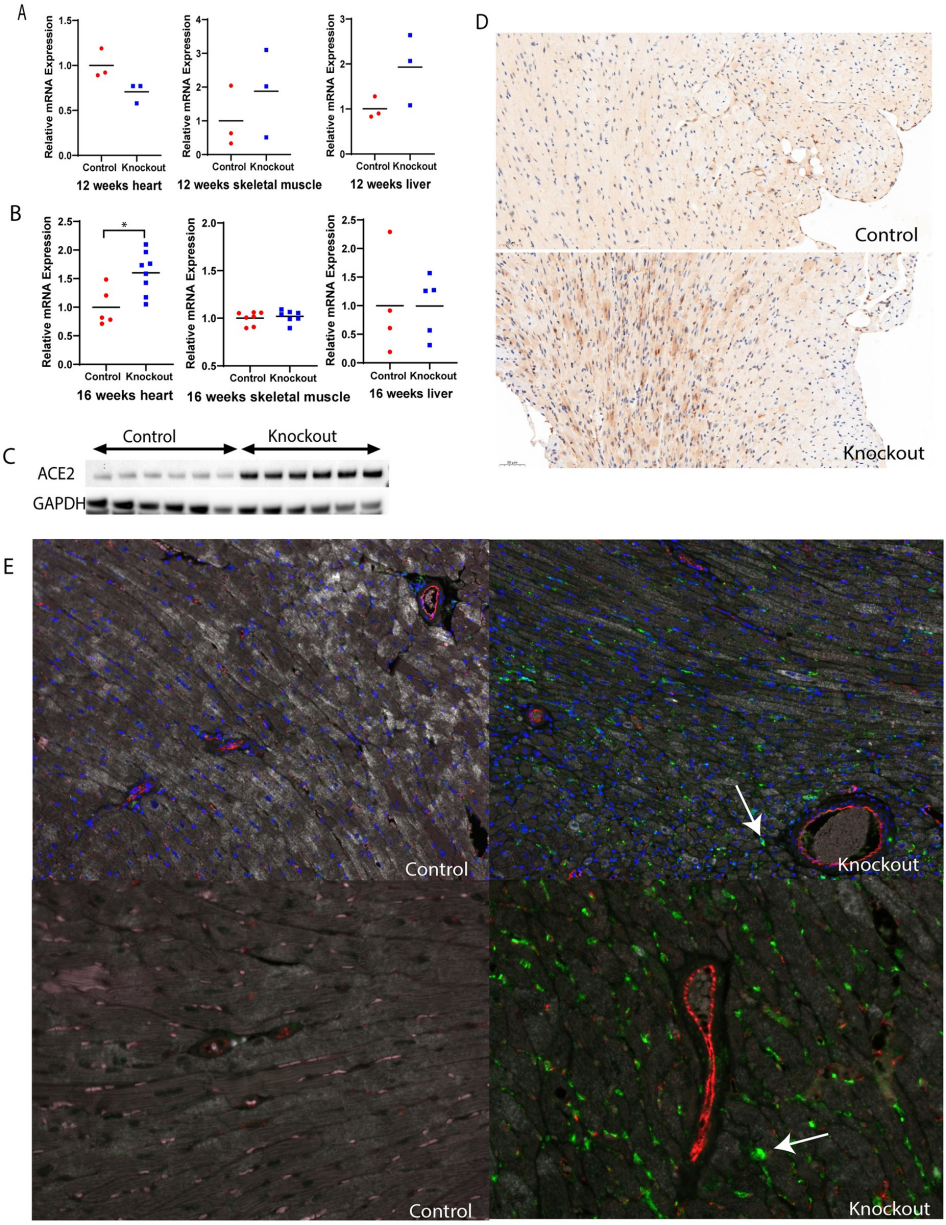
Angiotensin-Converting Enzyme 2 highly expressed in heart. (A) Ace2 gene expression in 12 weeks heart, skeletal muscle and liver. n = 3 mice per group. (B) Ace2 gene expression in 16 weeks heart, skeletal muscle and liver. n = 4–8 mice per group. (C) ACE2 high expressed in protein level in all knockout group mice. n = 6 mice per group. (D) Immunohistochemistry results of ACE2 staining. n = 3 mice per group. (E) Staining CD31 (red colour), CD68 (pink colour) and ACE2 (green colour) colocalization using confocal to acquire image, 20X magnification and 40X magnification. n = 3 mice per group. For (A-B), Mann-Whitney *U*-test was used to compare the control to the knockout groups. Significant levels were set to p < 0.05(*), p < 0.01(**), p < 0.001(***).

ACE2 is suggested to be involved in the regulation of the blood glucose levels (9), and we also found ACE2 were affected by mtDNA deficiency. For the time being there is a special interest in the ACE2 being the receptor of entry for the SARS-CoV-2 virus. We found it relevant to investigate the ACE2 expression in relation to mtDNA depletion and mitochondrial dysfunction. The *Ace2* gene was not altered in 12 weeks mice heart, skeletal muscle, or liver (Fig 5A). Interestingly, in heart tissue of 16 weeks old mTk2 KO mice high expression was detected of both the gene *Ace2* and the corresponding ACE2 protein (Fig 5B and 5C). The increased expression of ACE2 was only detected in heart tissue and not in skeletal muscle or liver of mTk2 KO mice (Fig 5B). The histopathology analysis confirmed that the mTk2 KO mice expressed higher levels of ACE2 protein in heart tissue (Fig 5D). We observed ACE2 signal in the myocardium, especially in privacular regions and areas with damaged cardiomyocytes. Multiplex staining for CD31, CD68 and ACE2 was performed to investigate whether endothelial cells and/or macrophages were involved in the expression of ACE2, and confocal microscopy confirmed that ACE2 signals appeared in cardiomyocytes (Fig 5E). We also observed that both control and knockout mice had a low basal level of ACE2 expression in the cardiomyocytes, but ACE2 was not observed in blood vessels or macrophages (Fig 5E).

## Discussion

We report a muscle specific TK2 knockout mouse strain suitable as a disease model for TK2 deficiency. The model presented showed a less severe phenotype as compared to the previously reported total TK2 knockout mouse, with a longer lifespan enabling future mechanistic and intervention studies. The genetic alteration proved to be specific for skeletal and heart muscle, that were affected with low mtDNA levels and pathology related to muscle tissue damage.

Interestingly, although the mitochondrial deficiency was restricted to muscle tissue of the mTk2 KO mice, we observed a rapid loss of the entire adipose tissue that progressed simultaneously with the decline of mtDNA in muscle tissue.

The mice were fed normally and had a normal liver function, but with deficient mitochondria in muscle tissue, they consumed the fat depots. In addition to depletion of adipose tissue also the skeletal muscle volume was decreased in the mTk2 KO mice. Metabolic stress can initiate catabolism of muscle and the decrease of muscle tissue may be a sign of amino acid consumption, that primarily produces energy through gluconeogenesis in the liver. In heart tissue the effect of dysfunctional muscle cells instead resulted in dilatation of the heart. The mtDNA depleted and thereby dysfunctional heart muscle cells were apparently not able to keep the continuous contractions and functions of the heart.

We have focused on the most obvious phenotypic alteration, that is the metabolic change with the loss of adipose tissue. In patients affected by TK2 deficiency the main reported symptom is myopathy with progressive muscle weakness leading to respiratory failure [12]. There are thus apparently both differences and similarities in the findings of our mTk2 KO mouse model and observations in affected patients. Whether fat deposits are affected in patients with TK2 deficiency is not clear in the literature.

Based on our observations we investigated how heart and skeletal muscle tissue utilized fatty acids, since mtDNA deficiency may have effects on fatty acid oxidation. AMPK is an important energy-sensing enzyme that monitors cellular energy status and functions by phosphorylation in response to low ATP/(AMP+ADP) ratios [13]. The mTk2 KO mice showed lower levels of an *Ampk* gene subunit expression in heart muscle tissue at 16 weeks of age, indicating that cardiomyocytes may have lost control of the ATP/(AMP+ADP) ratios at this late stage of disease. The gene encoding PGC-1α (*Ppargc1a*) is highly expressed in energy demanding tissues such as heart, brain, liver, skeletal muscle and kidney. Reduced expression of PGC-1α has been shown to be associated with heart failure in humans [14] and also with decreased expression of genes involved in ATP synthesis in mice heart tissue [15]. A shift from triglycerides to glucose in PGC-1α^−/−^ mice heart, was suggested to contribute to the development of cardiomyopathy. In the mTk2 KO mice, *Ppargc1a* was downregulated in heart tissue at 16 weeks of age, but not at 12 weeks, which corresponds to the observed deterioration of the heart muscle and abnormal cardiac muscle histology.

Lipoprotein lipase, encoded by the *Lpl* gene, is an enzyme that can both hydrolyze triglycerides and mediate lipoprotein uptake [16]. In skeletal muscle and heart tissue of 16 weeks old mTk2 KO mice the *Lpl* gene expression was down regulated, which may illustrate that the function of triglyceride catabolism was severely impaired. Interestingly, we observed alterations in liver tissue with increased expression of the *Lpl* gene in 16 weeks old mTk2 KO mice. And the LPL protein was detected at higher levels in both heart, skeletal muscle, and liver tissue. The lipid metabolism was not changed in liver according to the gene expression data except *Lpl* gene. Since the LPL can be secreted into the vessel and then relocated, it is possible that liver produced LPL that subsequently was relocated to take part in lipid metabolism in muscle tissue. Which means the LPL in the liver did not help transporting the triglyceride into the liver, but travel to the skeletal muscle and heart, carrying the tryglyceride to support essential energy requirement in muscle cells. This is in line with our hypothesis that liver is involved in the observed alterations of fat metabolism, that results in the scarce fat deposits found in the mTk2 KO mice.

The level of c-myc regulates cellular metabolism and is extensively studied in cancer cells [17,18]. Expression of the *c-myc* gene increased in both heart and skeletal muscle of the mTk2 KO mice at 16 weeks of age, as an additional sign of metabolic adaptation to the energy deficient state caused by mitochondrial dysfunction. Increased expression of the *c-myc* gene indicates a metabolic shift and the *c-myc* gene is highly expressed in embryos and tumor cells, which both depend on glycolysis [19,20]. Myc^(-/-)^ rat fibroblasts demonstrate low levels of glycolysis and oxidative phosphorylation, dysfunctional mitochondria and electron transport chain complexes [21]. The upregulation of *c-myc* in our study points to the importance of *c-myc* as a master regulatory gene for the metabolic adaptation, and thus the survival, of the mTk2 KO mice. Our study suggest involvement of *c-myc* also in mtDNA deficiency with its actions to increase the production of ATP and intermediates for macromolecule biosynthesis and to maintain cellular redox status.

A severe complication of the mtDNA depletion in heart tissue was a dilatation of the heart and we believe that the observed sudden death of the mice at ages 12–19 weeks was due to acute heart failure. Of particular interest was the increase of the ACE2 receptor, both at the gene expression and protein level, in the heart tissue of mtDNA deficient mice. ACE2 is a monocarboxypeptidase that degrades angiotensin I and angiotensin II into smaller peptides, which play an important role in the renin-angiotensin system and is expressed in several organs, such as the nasopharynx, lung, liver, kidney, and brain [22]. Enhancing ACE2 has a potential to protect cardiac cells though anti-hypertrophic, anti-fibrotic and antioxidant effects [23]. A decrease of insulin secretion and a cumulative disability of glucose tolerance was found in mice with ACE2 depletion when exposed to glucose [24]. Another interesting observation was ventricular tachycardia and sudden death in mice with increased expression of human ACE2 in cardiac cells [25]. The ACE2 receptor was specifically expressed in cardiomyocytes in our mTk2 KO mouse model and suggests a relationship between mitochondrial dysfunction and ACE2 expression. As an important energy producing organelle, the mitochondrion is deeply involved in glucose and fatty acid metabolim. Studies have shown that an activation of ACE2 led to enhanced glucose and lipid metabolism [26], indicating ACE2 to play a role in mitochondrial metabolim. Since ACE2 is the major SARS-CoV-2 entry-receptor [27], it is of special interest and its presence may influence the organs affected and the severity of the Covid19 disease. Our data demonstrate a link between mitochondrial DNA deficiency in heart tissue and a possible increased susceptibility to SARS-CoV-2 with risk of tissue damage and severe disease.

## Materials and methods

### Generation of muscle specific TK2 knockout mice

With homologous recombination, TK2 conditional knockout mice were generated that replaced original TK2 exon V with exon V adding two loxp sites, two flp sites, and a neo cassette. TK2^loxP^/TK2^loxP^ mice with neo cassette depletion were mated to heterozygous transgenic mice (+/Ckmm-cre). Double heterozygous (+/TK2 ^loxP^, +/Ckmm-cre) mice were backcrossed with TK2 ^loxP^/TK2 ^loxP^ strain to generate tissue specific knockout mice (TK2^-/-^, +/Ckmm-cre) (S.1). All animal experiments were approved and performed following the guidelines of the local ethical committee. The name of the ethical committee is: The Swedish Board of Agriculture; approval obtained in Linköping’s animal ethics committee, Linköping District Court. Approved number are 101–15, and 6487–2021. The study used cervical dislocation as a method to sacrifice the animal.

### Reagents

Rabbit polyclonal to ACE2 antibody (Abcam Inc, Cambridge, MA, USA, Ab15348), mouse monoclonal to MTCO1 (Abcam Inc, Cambridge, MA, USA, ab14705), anti CD68 (marker of macrophages) antibody (Life Technologies Europe,Bleiswijk, Netherlands, MA5-16363) from Thermo Fisher Scientific, anti CD31 (platelet endothelial cell adhesion molecule) antibody (Abcam Inc, Cambridge, MA, USA, ab28364) from Abcam, anti VDAC antibody (Santa Cruz Biotechnology, Inc. Dallas, Texas, U.S.A. sc390996), donkey anti rabbit with HRP (Santa Cruz Biotechnology, Inc. Dallas, Texas, U.S.A. sc-2313), Dako Polyclonal Rabbit anti-Mouse Immunoglobulins/HRP (Santa Clara, CA, United States, P0260). Dry ice, isopentane(2-methylbutane) from Sigma-Aldrich Sweden AB, Solkraftsvagen, Stockholm, 277258 CAS:78-78-4, cryostat embedding solution from Sakura Finetek Tissue, Torrance, CA, Tek 4583.

### Mitochondrial DNA copy number quantification

Total DNA was extracted from indicated tissues (Qiagen, DNeasy Blood & Tissue Kit 69506, Sofielundsvägen 4, Sollentuna, Sweden). The mtDNA copy number was determined with real-time PCR. Primers and probes for the mouse *mt-Nd1* gene (mitochondria encoded NADH dehydrogenase 1; forward primer: 5′-TCGACCTGACAGAAGGAGAATCA, reverse primer: 5′-GGGCCGGCTGCGTATT, probe: FAM-AATTAGTATCAGGGTTTAACG-TAMRA) and for single-copy mouse *Rpph1* gene (nuclear encoded ribonuclease P RNA component H1; forward primer: 5′-GGAGAGTAGTCTGAATTGGGTTATGAG, reverse primer: 5′-CAGCAGTGCGAGTTCAATGG, probe: FAM-CCGGGAGGTGCCTC-TAMRA) were designed for the quantification. For each DNA sample, the mitochondrial gene *mt-ND1* and the nuclear gene *Rpph1* was quantified separately. Standard curves were generated using known numbers of a plasmid containing one copy of each of the two mouse genes referred above. The plasmid was then diluted to the designated concentration to be used as standard curve reference. According to the standard curve, the number of copies from each gene was calculated for each sample, and the number of mtDNA copies per diploid nucleus was calculated according to the formula: mtDNA copies per diploid nucleus = 2 × (mt-*Nd1* gene copies/*Rpph1* gene copies).

### Gene expression determination

Total RNA was isolated using RNeasy Kit (Qiagen, 74106, Sofielundsvägen 4, Sollentuna, Sweden) for heart and liver, RNeasy Fibrous Tissue Mini Kit (50) (Qiagen, 74704, Sofielundsvägen 4, Sollentuna, Sweden) for skeletal muscle, and reverse transcript using a cDNA synthesis kit (Applied Biosystems, Life Technologies Corporation|Carlsbad, CA, 4368814). The target genes were measured by specific primers and the endogenous *Ppia* gene for heart tissue, the *Actb* gene for the skeletal muscle and liver tissue. Real-time PCR was performed by using KAPA SYBR^®^ Fast qPCR Master Mix (2X) Universal (Kapa Biosystems, Merck KGaA, Darmstadt, Germany, KM4602) in ABI 7500 Fast system (Applied Biosystems). The sequences of primers are listed in the supplemental data (S.4). The calculation was performed according to the delta delta method [28]. The figures are generated in software GraphPad Prism 8.

### Determination of fat volume with CT scan and muscle strength

We used PET CT scan analysis of 16 weeks old, sacrificed mice. Each group included three mice. The Analyze 12.0 software from AnalyzeDirect was used to determine the 3D fat and muscle volumes.

We measured the control and mTk2 KO mice muscle strength, each group included 5 mice. The animals were tested for muscle strength for a maximum of 2 minutes. They were allowed to hang from a grille vertically with four legs until it was observed that they had difficulties to hold on and then they let go. They were close to the ground and returned to their normal environment immediately. The control animals could easily stay for 2 min and then we let the mice free. We tested the mice every week between 4 to 16 weeks of age, and sacrificed the mice when they showed too low body weights or other conditions according to the ethical permit.

### Western blot, histology, COX/SDH staining and immunohistochemistry

At week 16, the mice were sacrificed, and organs were saved for histology and immunohistochemistry. Predetermined parts of the organs were fixed in 4% formaldehyde and cryomount (Histolab, Askim, Gothenburg, Sweden, 00890). The rest of the tissues were snap-frozen in liquid nitrogen and kept at—80°C for future use. The COX/SDH staining experiments followed the published protocol [29]. For the histology, COX/SDH staining, and immunohistochemistry, each group of controls and knockouts included three samples.

### Statistical analysis

Each individual mouse was measured as a biological replicate. For the real-time PCR, two or three technical replicates were performed for each biological replicate. Body weights, mtDNA copy number and fat volume were analyzed with unpaired two-tailed *t*-tests. The Mann-Whitney *U*-test was used to compare the non-parametric gene expression data. Significant level was set to p < 0.05.

## Supporting information

S1 Fig Generation of TK2 conditional knockout mice. Homologous recombination strategy of TK2 conditional knockout mice (A). breeding strategy of producing TK2 heart and skeletal muscle knockout mice (B). Genotyping results of heterozygous mice (1, 4) and homozygous mice (2) and wild type (3,5,6) (C). Genotyping results of heterozygous ckmm cre promoter (D). Tissue specific genotyping results of different organs (K: Kidney, L: Liver, H: Heart, S: Skeletal muscle) (E).
(DOCX)

S2 Fig Selection of adiposgenesis using the human myopathy data. The human myopathy data were used to synthesis the bar figure in https://maayanlab.cloud/Enrichr/, using human wiki pathway analysis, including control (GSM1054484, GSM1054485, GSM1054483), myopathy group (GSM1054462, GSM1054487, GSM1054481, GSM1054480, GSM1054479, GSM1054477, GSM1054476, GSM1054465, GSM1054464, GSM1054463).
(DOCX)

S3 Fig Gene expression data of 12 weeks heart and skeletal muscle, and 16 weeks heart. (A-C) 12 weeks mice heart (A), 16 weeks mice heart (B), and 12 weeks mice skeletal muscle (C) gene expression. n = 3–8 mice per group. Mann-Whitney test was used to compare the control to the knockout groups. Significant levels were set to p < 0.05(*), p < 0.01(**), p < 0.001(***).
(DOCX)

S1 File (XLSX)

S1 Raw images (PDF)
