# Supplementary material for: Mitochondrial dysfunction is associated with lipid metabolism disorder and upregulation of angiotensin-converting enzyme 2
Source: PLoS One. 2022 Jun 29;17(6):e0270418. doi: 10.1371/journal.pone.0270418 (PMC9242481; doi:10.1371/journal.pone.0270418)
Supplement: S1 Fig — Homologous recombination strategy of TK2 conditional knockout mice (A). breeding strategy of producing TK2 heart and skeletal muscle knockout mice (B). Genotyping results of heterozygous mice (1, 4) and homozygous mice (2) and wild type (3,5,6) (C). Genotyping results of heterozygous ckmm cre promoter (D). Tissue specific genotyping results of different organs (K: Kidney, L: Liver, H: Heart, S: Skeletal muscle) (E). (DOCX) [file pone.0270418.s001.docx]

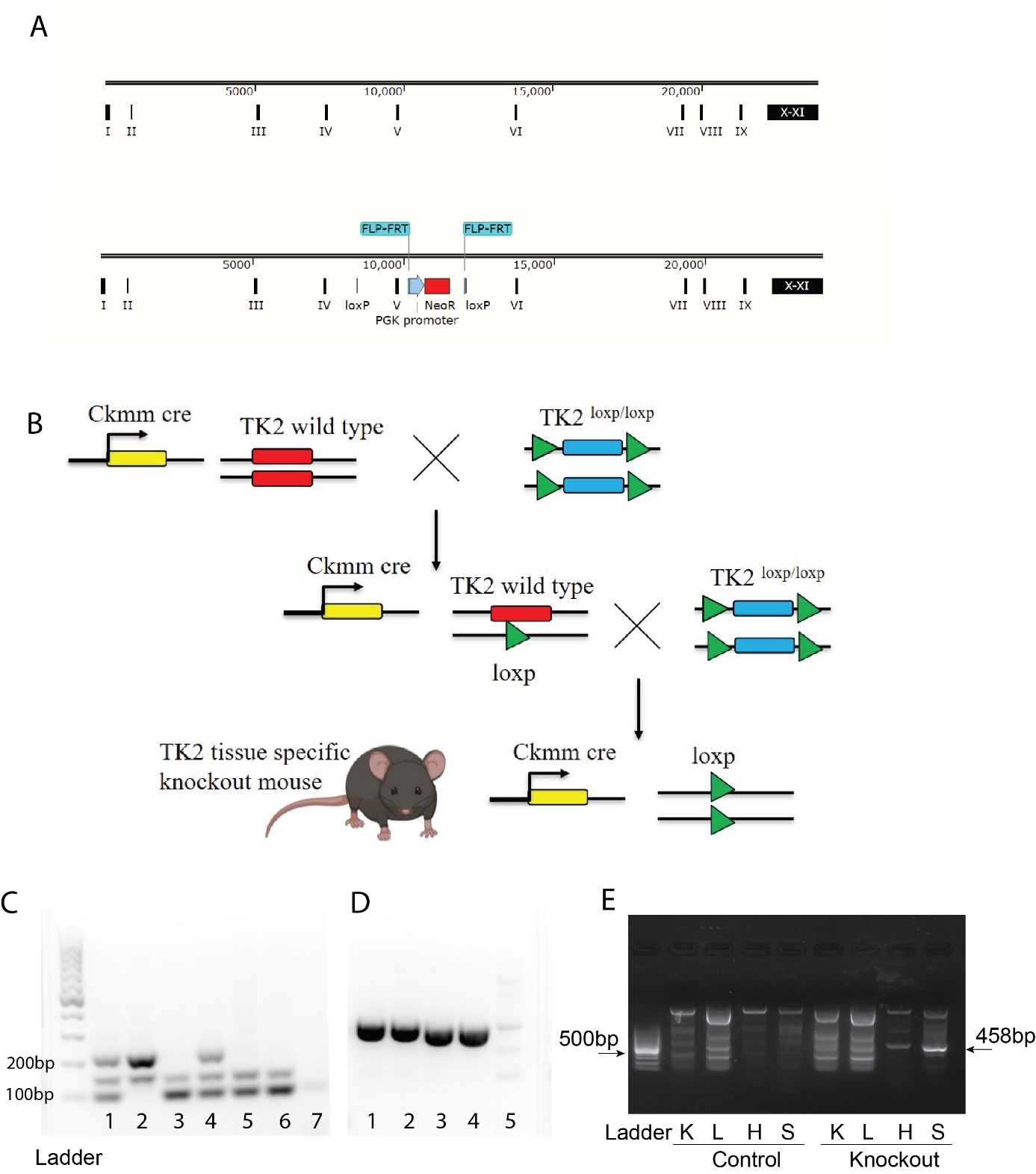


**S1 Fig Generation of TK2 conditional knockout mice**

homologous recombination strategy of TK2 conditional knockout mice (A). breeding strategy of producing TK2 heart and skeletal muscle knockout mice (B). Genotyping results of heterozygous mice (1, 4) and homozygous mice (2) and wild type (3,5,6) (C). Genotyping results of heterozygous ckmm cre promoter (D). Tissue specific genotyping results of different organs (K: kidney, L: liver, H: heart, S: skeletal muscle) (E).
