# Supplementary material for: Mitochondrial dysfunction is associated with lipid metabolism disorder and upregulation of angiotensin-converting enzyme 2
Source: PLoS One. 2022 Jun 29;17(6):e0270418. doi: 10.1371/journal.pone.0270418 (PMC9242481; doi:10.1371/journal.pone.0270418)
Supplement: S2 Fig — The human myopathy data were used to synthesis the bar figure in https://maayanlab.cloud/Enrichr/, using human wiki pathway analysis, including control (GSM1054484, GSM1054485, GSM1054483), myopathy group (GSM1054462, GSM1054487, GSM1054481, GSM1054480, GSM1054479, GSM1054477, GSM1054476, GSM1054465, GSM1054464, GSM1054463). (DOCX) [file pone.0270418.s002.docx]

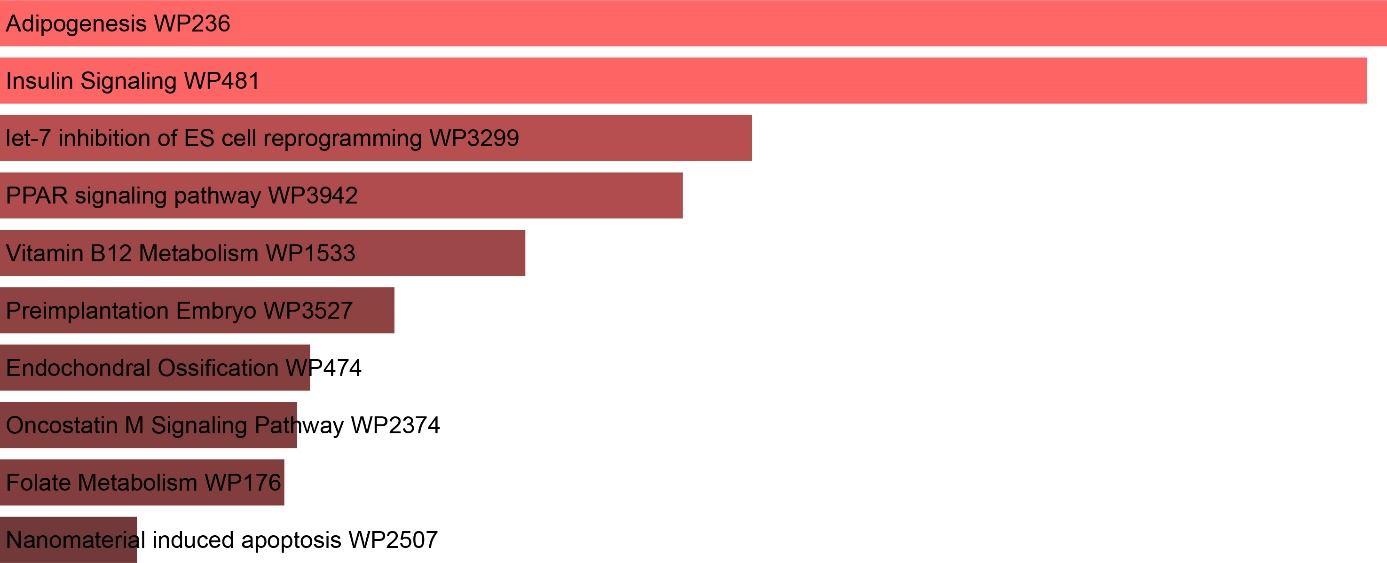


**S2 Fig**, **selection of adiposgenesis using the human myopathy data**

The human myopathy data were used to synthesis the bar figure in https://maayanlab.cloud/Enrichr/, using human wiki pathway analysis, including control (GSM1054484, GSM1054485, GSM1054483), myopathy group (GSM1054462, GSM1054487, GSM1054481, GSM1054480, GSM1054479, GSM1054477, GSM1054476, GSM1054465, GSM1054464, GSM1054463)
