# Supplementary material for: Mitochondrial dysfunction is associated with lipid metabolism disorder and upregulation of angiotensin-converting enzyme 2
Source: PLoS One. 2022 Jun 29;17(6):e0270418. doi: 10.1371/journal.pone.0270418 (PMC9242481; doi:10.1371/journal.pone.0270418)
Supplement: S3 Fig — (A-C) 12 weeks mice heart (A), 16 weeks mice heart (B), and 12 weeks mice skeletal muscle (C) gene expression. n = 3–8 mice per group. Mann-Whitney test was used to compare the control to the knockout groups. Significant levels were set to p < 0.05(*), p < 0.01(**), p < 0.001(***). (DOCX) [file pone.0270418.s003.docx]

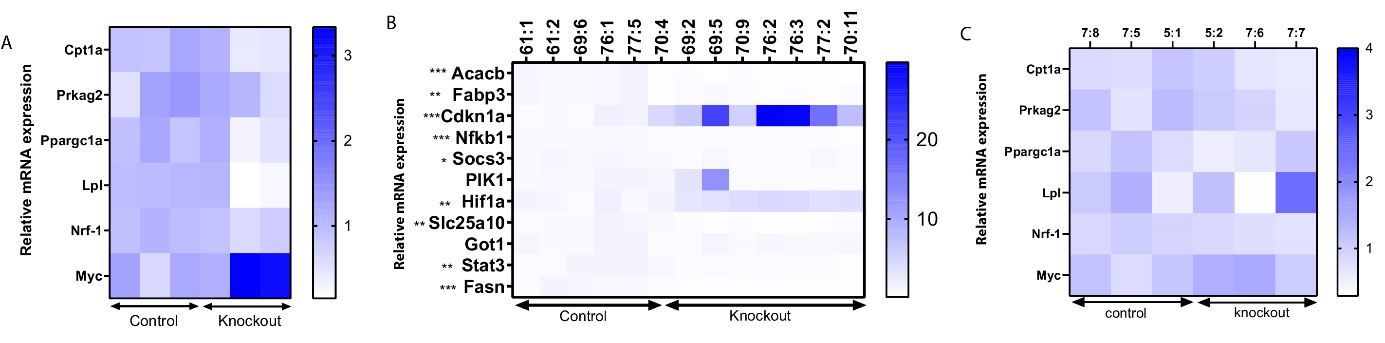


**S3 Fig**, gene expression data of 12 weeks heart and skeletal muscle, and 16 weeks heart.

(A-C) 12 weeks mice heart (A), 16 weeks mice heart (B), and 12 weeks mice skeletal muscle (C) gene expression. n = 3-8 mice per group. Mann-Whitney test was used to compare the control to the knockout groups. Significant levels were set to p < 0.05(*), p < 0.01(**), p < 0.001(***).
