# Supplementary material for: Mitochondrial dysfunction is associated with lipid metabolism disorder and upregulation of angiotensin-converting enzyme 2
Source: PLoS One. 2022 Jun 29;17(6):e0270418. doi: 10.1371/journal.pone.0270418 (PMC9242481; doi:10.1371/journal.pone.0270418)
Supplement: S1 Raw images — (PDF) [file pone.0270418.s005.pdf]

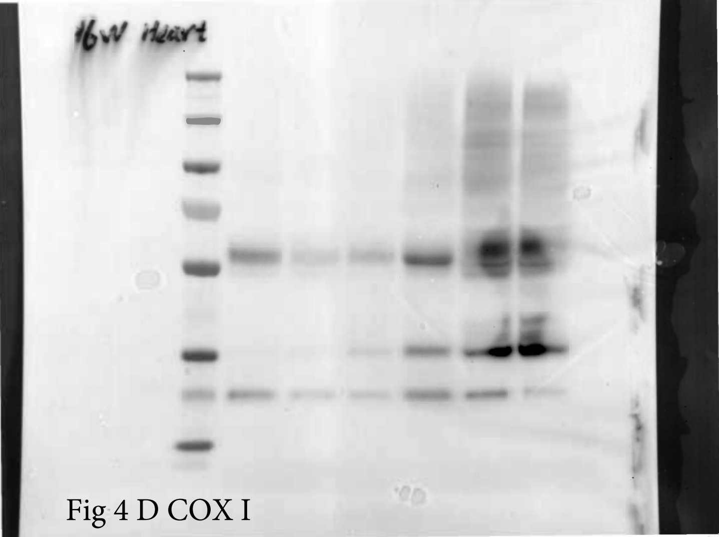

Fig 4 D COX I

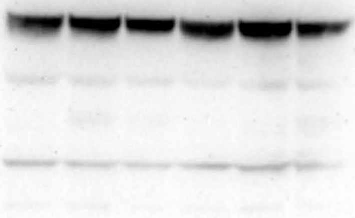

Fig 4 D SDHA

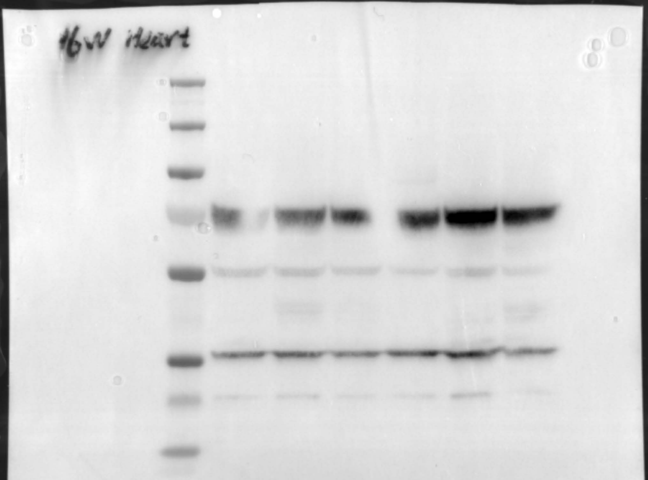

Fig 4 D VDAC

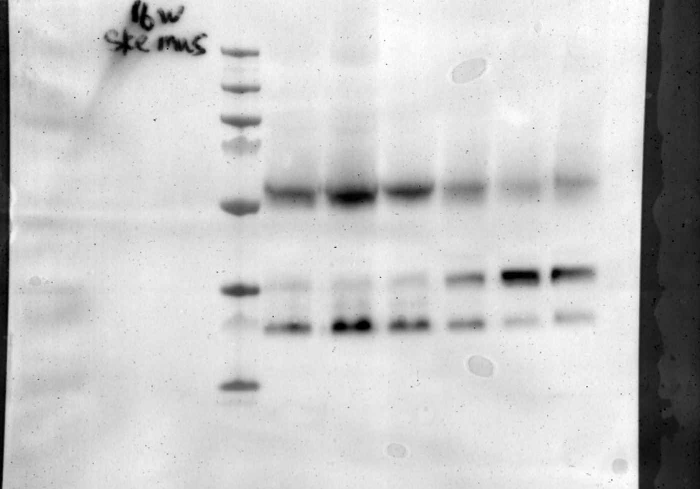

Fig 4 E COX I

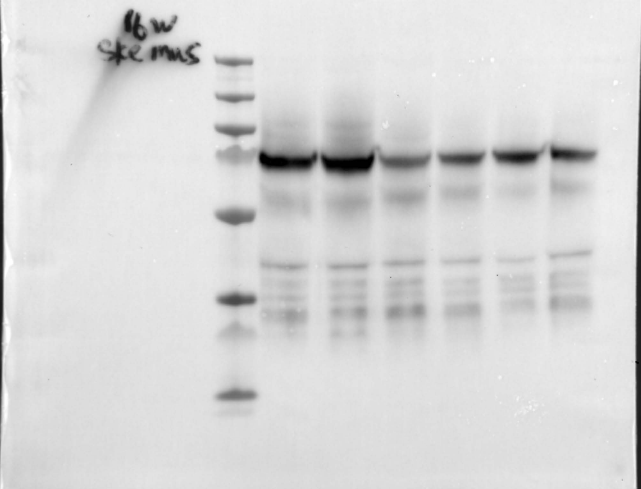

Fig 4 E SDHA

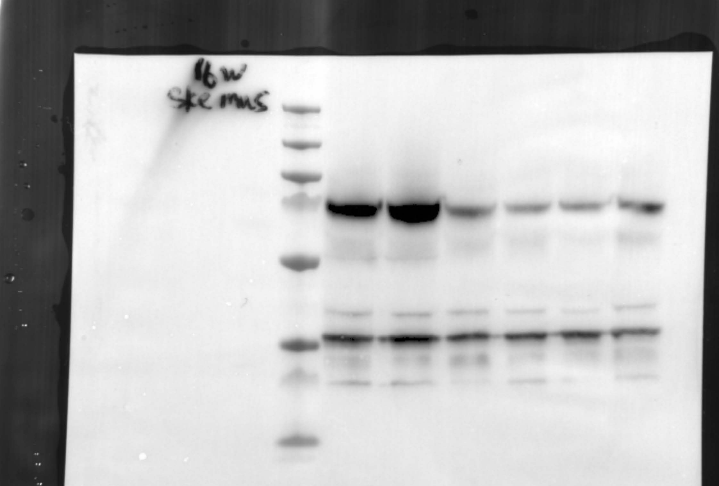

Fig 4 E VDAC

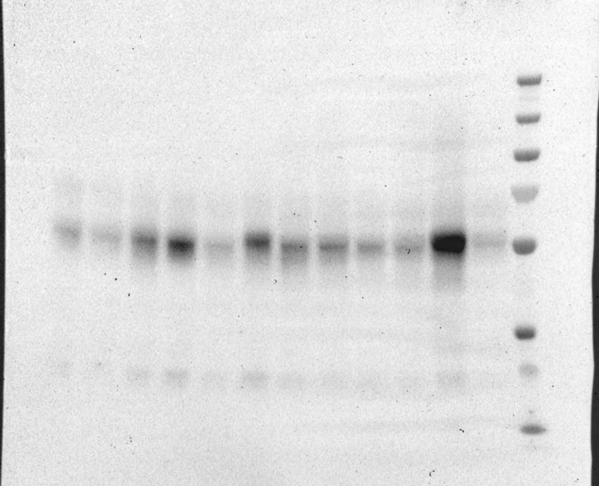

Fig 4 F heart LPL

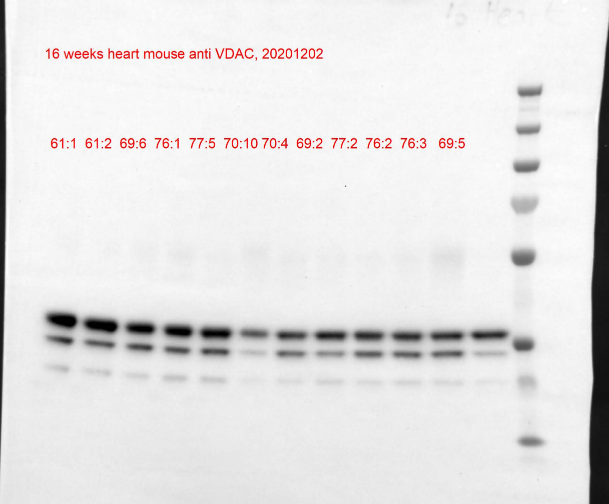

Fig 4 F heart VDAC

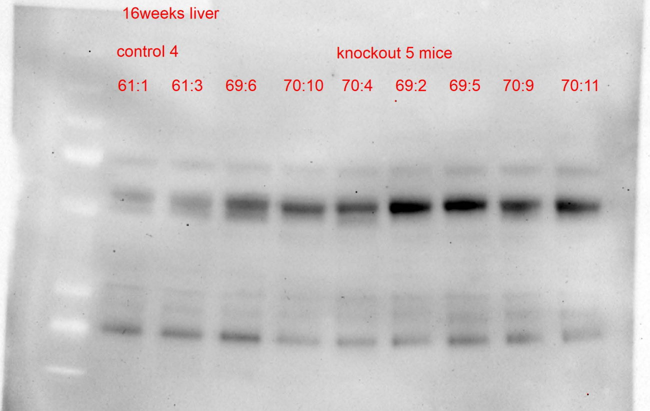

Fig 4 F liver LPL

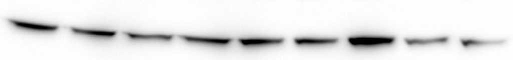

Fig 4 F liver VDAC

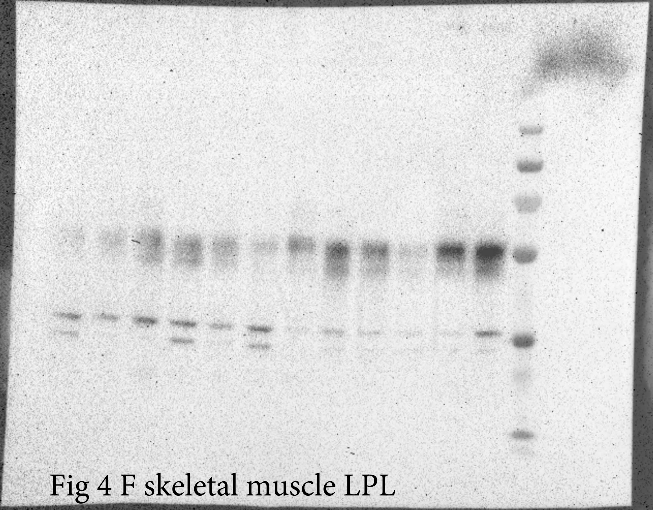

Fig 4 F skeletal muscle LPL

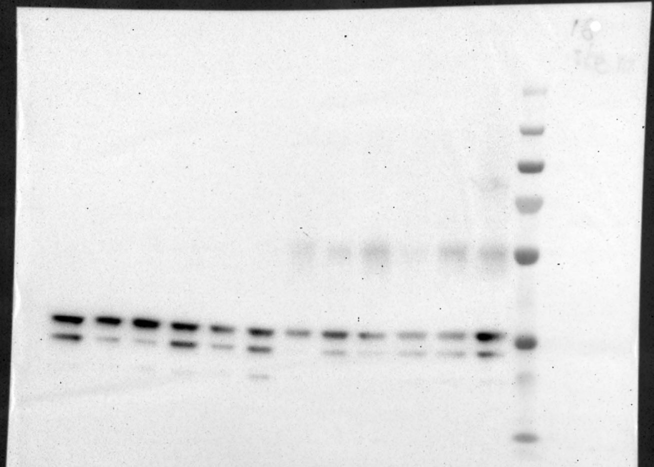

Fig 4 F skeletal muscle VDAC

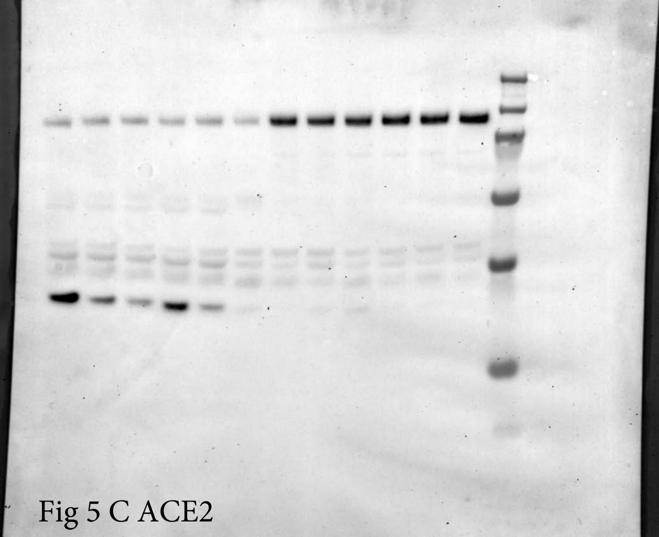

Fig 5 C ACE2

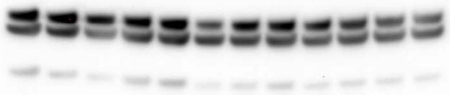

Fig 5 C GAPDH

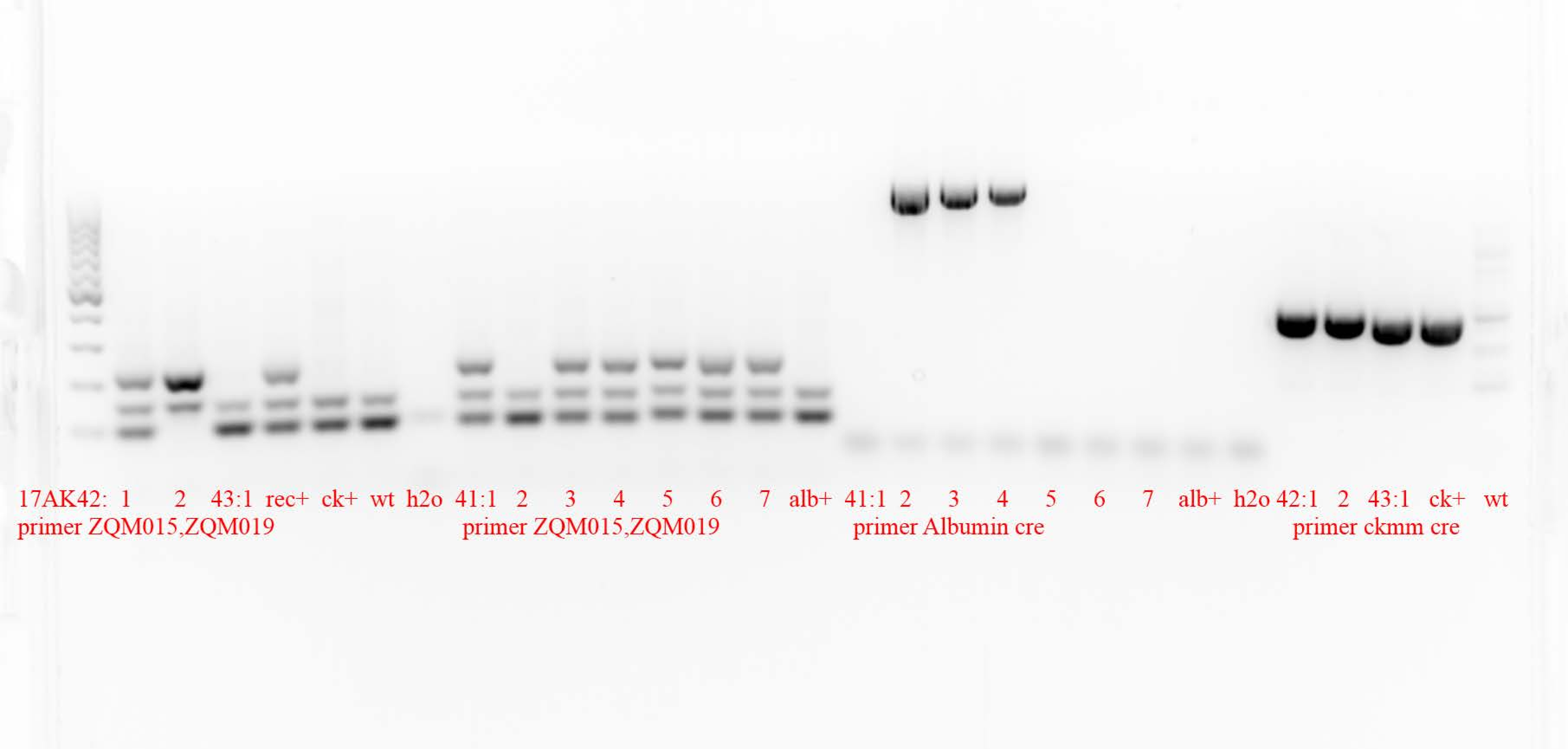
17AK42: 1 2 43:1 rec+ ck+ wt h2o 41:1 2 3 4 5 6 7 alb+ 41:1 2 3 4 5 6 7 alb+ h2o 42:1 2 43:1 ck+ wt
primer ZQM015,ZQM019
primer ZQM015,ZQM019
primer Albumin cre
primer ckmm cre

S1 Fig C, D

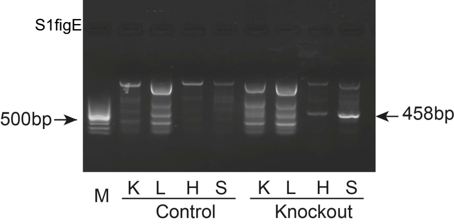
S1figE
500bp
458bp
M
K L H S
Control
K L H S
Knockout
